# Stimulating ideas for heart regeneration: the future of nerve-directed heart therapy

**DOI:** 10.1186/s42234-019-0024-0

**Published:** 2019-06-26

**Authors:** Emma B. Brandt, S. Janna Bashar, Ahmed I. Mahmoud

**Affiliations:** Department of Cell and Regenerative Biology, University of Wisconsin-Madison School of Medicine and Public Health, 1111 Highland Ave, Room 4557, Madison, WI 53705 USA

**Keywords:** Cardiac regeneration, Cholinergic nerves, Heart disease, Inflammation, Vagus nerve stimulation (VNS)

## Abstract

Ischemic heart disease is the leading cause of death worldwide. The blockade of coronary arteries limits oxygen-rich blood to the heart and consequently there is cardiomyocyte (CM) cell death, inflammation, fibrotic scarring, and myocardial remodeling. Unfortunately, current therapeutics fail to effectively replace the lost cardiomyocytes or prevent fibrotic scarring, which results in reduced cardiac function and the development of heart failure (HF) in the adult mammalian heart. In contrast, neonatal mice are capable of regenerating their hearts following injury. However, this regenerative response is restricted to the first week of post-natal development. Recently, we identified that cholinergic nerve signaling is necessary for the neonatal mouse cardiac regenerative response. This demonstrates that cholinergic nerve stimulation holds significant potential as a bioelectronic therapeutic tool for heart disease. However, the mechanisms of nerve directed regeneration in the heart remain undetermined. In this review, we will describe the historical evidence of nerve function during regeneration across species. Specifically, we will focus on the emerging role of cholinergic innervation in modulating cardiomyocyte proliferation and inflammation during heart regeneration. Understanding the role of nerves in mammalian heart regeneration and adult cardiac remodeling can provide us with innovative bioelectronic-based therapeutic approaches for treatment of human heart disease.

## Background

**Fig. 1:**
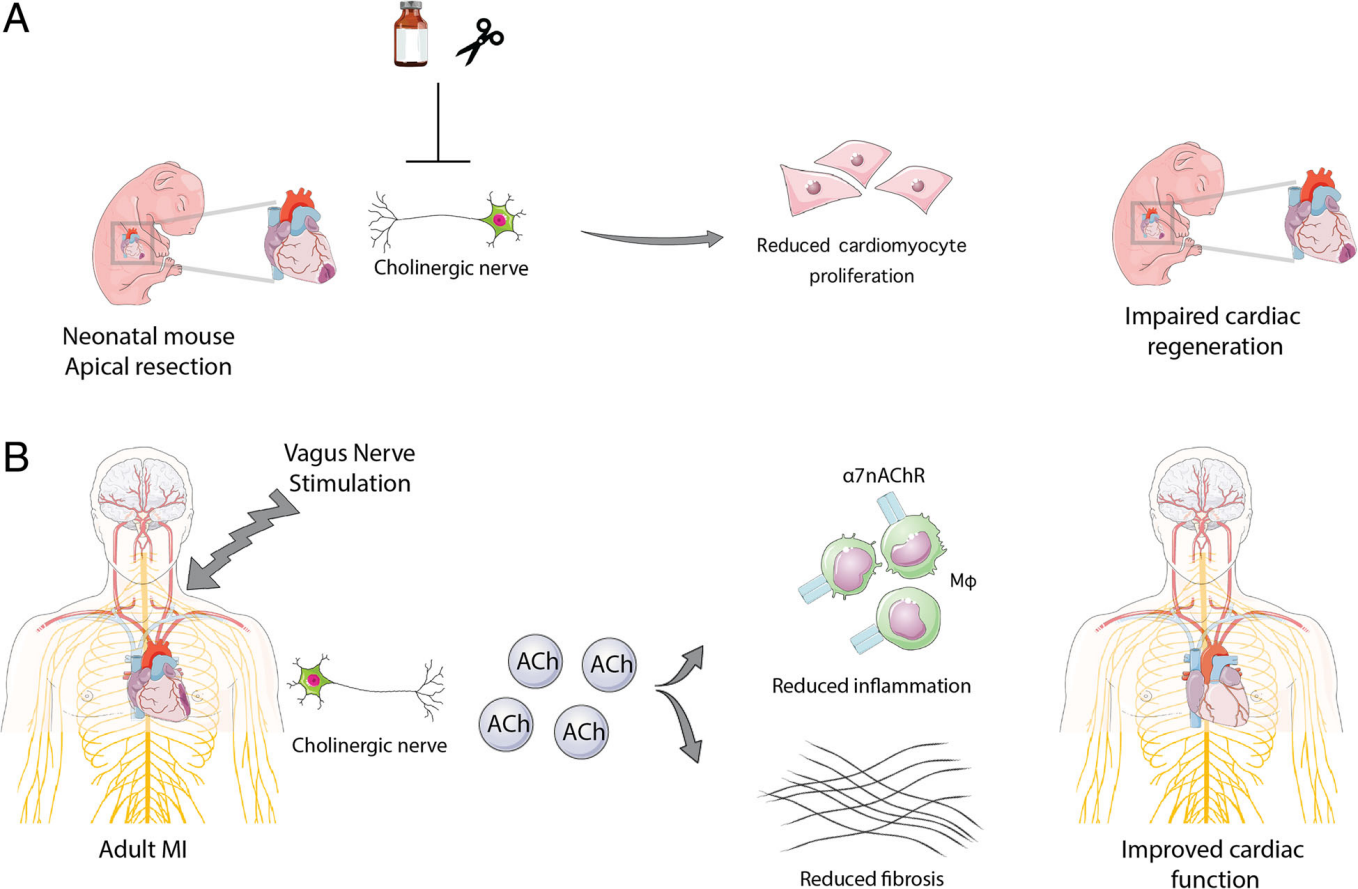
Cholinergic nerve signaling promotes neonatal heart regeneration and adult heart remodeling following cardiac injury. **a** The neonatal mouse heart is capable of regenerating following injury, and this regenerative response was demonstrated to be dependent on cholinergic nerve signaling for proper heart regeneration. Pharmacological and mechanical blocking of cholinergic nerves resulted in reduced cardiomyocyte proliferation and failure to regenerate. **b** Adults with chronic heart failure treated with vagus nerve stimulation showed some improvements in heart function although these clinical studies were preliminary. The mechanisms behind the beneficial role of vagus nerve stimulation in cardiac repair are still not fully understood. However, it is suggested that the beneficial effect of VNS is partly mediated via increased ACh signaling in macrophages through binding to the *α* 7nAChR. This ultimately results in decreased secretion of pro-inflammatory cytokines and subsequent decrease in the heart’s fibrotic remodeling response

In this review, our goal is to highlight the evolutionarily conserved role of nerve signaling in promoting regeneration following injury across multiple tissues and different species. More importantly, we highlight the importance of cholinergic nerve signaling in regulating neonatal mouse heart regeneration, where ablation of cholinergic nerve signaling either pharmacologically or mechanically resulted in impaired cardiac regeneration and revealed a previously unappreciated role for nerves in cardiac regeneration (Fig. 1a). In addition, we discuss the intersection between cholinergic nerve signaling and the immune response during cardiac regeneration in the neonatal heart, as well as during cardiac repair and remodeling in the adult heart. The cholinergic anti-inflammatory pathway holds special interest for bioelectronic medicine since vagus nerve stimulation (VNS) is currently used in the clinic for treatment of immune disorders, epilepsy and depression (Ben-Menachem et al. 2015). Finally, we summarize the results of the VNS clinical trials to promote cardiac remodeling in adults with heart failure. Collectively, these findings as well as future studies aimed at elucidating the mechanisms of cholinergic nerve stimulation of cardiomyocyte proliferation, inflammation and heart regeneration have important therapeutic potential for bioelectronic methods to stimulate adult heart repair.

### Nerve-dependent regeneration across species

Nerve dependent regeneration has been observed in multiple tissues across different species (Kumar and Brockes 2012). Nerve dependent limb regeneration has been thoroughly characterized in salamanders (such as the newt and axolotl) (Farkas and Monaghan 2017), as they demonstrate a remarkable capacity of regenerating their limbs following amputation (Kumar and Brockes 2012; Kumar et al. 2007). Salamander limb regeneration occurs through the proliferation of the blastema, which are mesenchymal growth zone progenitors recruited to the wound plane epithelium after amputation (Butler and O'Brien 1942). The ability of the blastema to replace the amputated limb has been demonstrated to be specifically dependent on proper nerve function (Kragl et al. 2009; Kumar et al. 2007). Denervation prior to amputation does not affect initial blastema recruitment but does lead to a regression of the blastema and an incomplete regeneration of the amputated limb (Brockes 1984; Mescher and Tassava 1975). This suggests that nerves stimulate and maintain the regenerative mechanisms during salamander limb regeneration. Further studies showed that during newt limb regeneration, the re-innervated nerve supply releases Anterior Gradient (nAG) proteins from Schwann cells. nAG proteins when bound to blastema cell surface receptor, Prod1, function as a growth factor that allows the dissociated blastema to proliferate (Kumar et al. 2007). Therefore, nerve derived factors may be directly or indirectly stimulating cell proliferation during limb regeneration.

Nerve-driven regenerative programs have been documented in other higher-level vertebrates, but the mechanisms are less understood. Regeneration of the catfish barbels, a taste sensory organ, depends on nerve supply, where denervation causes regression of the barbel regeneration (Kamrin and Singer 1955).

In zebrafish, sensory and motor denervation following adult zebrafish pectoral fin amputation can result in formation of the wound epithelium, however there is absent or impaired blastema expansion and this was associated with impaired regeneration (Simoes et al. 2014). Timing of reinnervation also appears to be significant for regenerative capacity, as denervation after blastema formation did not affect regeneration following amputation. The effect of denervation on zebrafish blastema expansion during pectoral fin regeneration was attributed to failure of the mesenchymal cells to overcome G2/M cell cycle arrest.

Furthermore, recent evidence suggests a requirement for nerve function in mammalian regenerative responses. Mice have demonstrated the capacity to regenerate digit tips after amputation (Takeo et al. 2013), and denervation can inhibit digit tip regeneration in mice (Johnston et al. 2016). Similarly, fetal lambs that were denervated and subjected to incisional wounds resulted in scar formation, and their open wounds failed to heal, suggesting that nerves play a role in the early phase of the regenerative response of fetal lamb wound healing (Stelnicki et al. 2000). More importantly, clinical cases of denervation in humans such as spinal cord injury and diabetic neuropathy, are associated with impaired cutaneous wound healing in denervated limbs (Rappl 2008) (Galkowska et al. 2006). Interestingly, a recent study by Carr et al. demonstrated the existence of mesenchymal-like precursor cells in adult mouse peripheral nerves that were suggested to contribute to mammalian tissue regeneration (Carr et al. 2019).

Furthermore, proper nerve function has also been implicated for efficient regeneration in invertebrates. Planaria are known for robust whole body regeneration, however this regenerative capacity is disrupted following denervation, which results in ectopic patterning of blastema regeneration (Cebria and Newmark 2007). Similarly, arm regeneration in starfish is dependent on the radial nerves which radiate from the oral nerve ring. Transection of the oral nerve ring or the radial nerve is sufficient to block arm regeneration in starfish (Huet 1975). Thus, nerve function represents an evolutionarily conserved regenerative program across species that can potentially be harnessed for promoting tissue repair and regeneration in non-regenerating adult mammalian tissues. However, the role of nerve-guided regenerative programs in mammals remains incompletely understood.

### Cholinergic nerve regulation of heart regeneration

Previously, strategies for promoting cardiac regeneration following injury focused primarily on identifying cardiac progenitor populations that can differentiate to replace the lost cardiomyocytes (CM) following injury, yet these isolated cells showed little capacity to differentiate into cardiomyocytes or promote any meaningful cardiac repair (Le and Chong 2016). Interestingly, recent evidence demonstrated that cardiomyocyte turnover occurs in the adult human heart however at very low levels, which are insufficient to replace the massive loss of cardiac tissue following injury (Bergmann et al. 2015). In contrast, adult zebrafish hearts are capable of mounting an endogenous regenerative program and are able to completely regenerate their myocardium through cardiomyocyte proliferation following ventricular resection (Poss et al. 2002). This regenerative potential was found to be conserved in the mammalian heart, but only for a brief time after birth in both neonatal mice and pigs (Ye et al. 2018; Zhu et al. 2018). These models of endogenous cardiac regeneration revealed that regeneration is mediated by the proliferation of the pre-existing cardiomyocytes. This new paradigm provides a platform to investigate the endogenous regenerative responses to injury that could help promote adult cardiomyocyte proliferation and heart regeneration (Porrello et al. 2011; Poss et al. 2002).

Recently, we have shown that cholinergic nerve function is essential for complete heart regeneration in both adult zebrafish and neonatal mice (Mahmoud et al. 2015). Transgenic zebrafish that overexpress the neurorepellent *sema3aa* showed reduced cardiomyocyte proliferation and incomplete regeneration following ventricular resection (Mahmoud et al. 2015). Specifically, using adult zebrafish and neonatal mice, we identified that inhibition of cholinergic nerve function, either by pharmacological or mechanical blockade, impaired cardiac regeneration following heart injury. The consequential loss of cholinergic signaling in neonatal mice led to a reduction in cardiomyocyte proliferation, which was evident in the reduced expression of cell cycle genes, as well as a reduced expression of the growth factors Nrg1 and Ngf. Injection of NRG1 and NGF recombinant proteins partially rescued the impaired regeneration following denervation. Furthermore, transcriptional profiling of regenerating and denervated hearts following vagotomy demonstrated a reduced inflammatory response following denervation. This suggested that cholinergic nerve signaling regulates cardiomyocyte cell cycle partly through modulation of growth factors and inflammation. Collectively, these results demonstrated that cholinergic innervation is essential for regulation of mammalian heart regeneration (Fig. 1a); however; the mechanisms by which cholinergic signaling regulates cardiomyocyte proliferation and heart regeneration requires further investigation.

### Inflammatory response during cardiac regeneration

A proper inflammatory response is one of the earliest events in regeneration and has proven essential in numerous tissues and species (Aurora and Olson 2014). Macrophages have been suggested to play a critical role in the regeneration process, since macrophage depletion in the salamander limb impaired their ability to regenerate unless the macrophages were restored (Godwin et al. 2013). Similarly, in mammals, depletion of macrophages in a neonatal myocardial infarction (MI) model impaired their heart regeneration abilities and emphasized the critical role that macrophages and monocytes play in the initial stages of regeneration (Aurora et al. 2014; Leor et al. 2016). The inflammatory response following an MI in the adult heart is characterized by a biphasic wave of macrophage recruitment where M1 (Ly6^hi^) macrophages (CCR2+) accumulate and act to scavenge any debris and create a pro-inflammatory environment. The M2 (Ly6^lo^) macrophages (CCR2-) follow to promote extracellular matrix (ECM) deposition and fibrotic scar formation, angiogenesis, and promoting anti-inflammatory cytokines like IL-10 (Aurora et al. 2014; Shiraishi et al. 2016). Further work on macrophages residing within the heart revealed an embryonic-derived population of macrophages that were established in the tissue prior to birth and continue to proliferate throughout the adult life. These embryonic or tissue-derived macrophages are characterized by MHC-II^high^ (major histocompatibility complex II)/CCR2- expression. Other post-natal recruited cardiac macrophages are MHC-II^low^/CCR2-, and a third population that are CCR2+ arise from definitive hematopoiesis (Honold and Nahrendorf 2018).

These tissue-resident macrophages were lost in the infarcted area but were able to proliferate and remain active in the border zone of the injury. Interestingly, single cell RNA-Seq analysis revealed that monocyte-derived macrophages were capable of adopting a transcriptional signature very similar to the embryonic-derived macrophages near the injured area. The depletion of these resident macrophages caused impaired healing and remodeling surrounding the infarct area (Dick et al. 2019). Additionally, a recent paper further characterized the mechanistic differences between the roles of CCR2- macrophages and the CCR2+ macrophages following an MI (Bajpai et al. 2019). The authors utilized diphtheria toxin to either selectively ablate CCR2+ macrophages, or to destroy tissue-derived CCR2- macrophages. This allowed the authors to address what roles these distinct macrophage populations play following injury in the neonatal and adult hearts. RNA-sequencing revealed up to 7 other additional subtypes of macrophages within the adult heart. Additionally, loss of CCR2- macrophages led to increased inflammation and increased monocyte and macrophage recruitment, whereas loss of CCR2+ macrophages reduced the inflammatory response following injury, which resulted in a significant impact on enhancing or reducing cardiac function following MI, respectively (Bajpai et al. 2019).

The immune response mounted following an MI plays a critical role in regulating cardiac tissue regeneration. Neonatal mice retain their ability for heart regeneration during the first week of life but this is quickly lost as the mice mature (Porrello et al. 2011). It has been demonstrated that the neonatal mouse regenerative capacity is more dependent on their innate immune system, showing decreased leukocyte invasion and increased IL-10 levels, which drives healing towards regeneration instead of fibrosis. Adult healing responses seem to favor the faster assembly of a fibrotic scar in order to maintain the integrity and stiffness of the cardiac structure following an injury (Sattler and Rosenthal 2016). Sattler and Rosenthal review this concept by comparing the immune systems between the neonatal and adult hearts following an MI (Sattler and Rosenthal 2016). It can be concluded that while increased inflammation in the heart can be more harmful than beneficial for proper repair, having no inflammatory response proved to be detrimental (Aurora and Olson 2014; Aurora et al. 2014). Therefore, it needs to be emphasized that there is a carefully balanced inflammatory response which allows the mammalian heart to regenerate. This could be due to the immature immune system in the neonate that results in reduced fibrosis and less damage from inflammation as compared to the adult heart. Collectively, these results reveal an important role for inflammation during heart regeneration, and thus promoting a reparative inflammatory profile is necessary to achieve proper regeneration.

### Cholinergic anti-inflammatory pathway

One important intersection between inflammation and nerve-directed regeneration is the cholinergic nervous system. The cholinergic anti-inflammatory pathway is a physiological mechanism in which acetylcholine released from cholinergic nerves acts on nicotinic acetylcholine receptors (nAChR) on macrophages, monocytes, lymphocytes, and other components of the immune system to modulate the production of pro-inflammatory cytokines (Pavlov et al. 2018). The receptor α7nAChR was found to be responsible for this regulation of cytokine production via a post-transcriptional inhibition of pro-inflammatory cytokines such as TNF or through the inhibition of NFκB (Rosas-Ballina et al. 2011; Wang et al. 2003). This inhibition of a pro-inflammatory environment and subsequent regulation of long-term chronic inflammation is dependent on continued cholinergic signaling. We previously highlighted the critical role that cholinergic nerves play in zebrafish and neonatal mouse heart regeneration (Fig. 1a) and briefly explored how immune responses change following an MI in both neonatal and adult mice. Our previous work has demonstrated that inhibition of cholinergic nerve function impairs cardiac regeneration (Mahmoud et al. 2015), but these effects on the neonatal immune system are still unclear. Thus, we currently have little understanding of the role of the cholinergic anti-inflammatory pathway in the regenerating hearts of neonates, although stimulating this pathway has been utilized in various clinical settings for autoimmune disorders, depression, and MI in adults (Pavlov and Tracey 2017). Studies of the cholinergic anti-inflammatory pathway in adult heart failure have typically relied on cholinergic nerve stimulation as a treatment to reduce inflammation by increasing cholinergic signaling. Administration of the cholinesterase inhibitor pyridostigmine in rats following MI stimulated the recruitment of more M2 macrophages to the infarct site and the surrounding area (Bezerra et al. 2017; Rocha et al. 2016). This recruitment of M2 macrophages increased the levels of anti-inflammatory cytokines such as Il-10, which led to a reduction in the inflammatory response following MI. It is worth noting that cardiomyocytes can produce their own ACh and contribute to non-neuronal cholinergic signaling, however for the scope of this review we will focus on the cholinergic nerves solely (Rocha-Resende et al. 2012; Saw et al. 2018).

Initial reports of the cholinergic anti-inflammatory pathway in the cardiovascular system for clinical use investigated the protective effects of vagus nerve stimulation (VNS) in rats, dogs, rabbits, and pigs in chronic heart failure (Ando et al. 2005; Calvillo et al. 2011; Hamann et al. 2013; Nuntaphum et al. 2018; Uemura et al. 2010; Vaseghi et al. 2017). VNS demonstrated a wide range of effects on the heart following injury, including anti-arrhythmogenic effects following ischemia in rats and rabbits (Ando et al. 2005; Brack et al. 2011; Wu et al. 2017), reduced inflammation and infarct size (Calvillo et al. 2011; Wu et al. 2017), prevention of remote vascular disfunction (Zhao et al. 2013), improved redox status after MI (Shinlapawittayatorn et al. 2014; Tsutsumi et al. 2008), and has been demonstrated to be tolerable in humans with some efficacy in advanced heart failure states (Klein and Ferrari 2010; Schwartz et al. 2008).

This beneficial effect after MI was proposed to be mediated partly through changes in TNF- *α* signaling although the exact mechanisms remain controversial. One study by Katare et al., in a mouse model showed that VNS resulted in an increase in TNF- *α* that they suggested had a cardiac protective role by signaling through the TNFR2 receptor instead of TNFR1 (Katare et al. 2010). In contrast, another study in rats utilized VNS following an MI and showed that TNF- *α* levels decreased, although they also showed the same increase in TNFR2 expression as the previous group (Kong et al. 2011). Together, these disparate results illustrate a need for more mechanistic studies to identify the impact of VNS on the inflammatory response in the adult and neonate heart to better understand its promising beneficial effects.

### Vagus nerve stimulation (VNS) as a bioelectronic therapeutic tool for heart disease

Human trials using vagus nerve stimulation for treatment of heart failure (HF) have shown mixed results thus far. The first study reported had only 8 male patients with chronic heart failure, who were fitted with an implantable CardioFit (﻿BioControl Medical Ltd., Yehud, Israel) neuro-stimulator with electrodes that measured heart rate and would cease stimulation if the heart rate dropped below 55 bpm. The average intensity of the stimulation was 4.3 ± 0.9 mAmp with few side effects including pain, cough, and voice alteration. Patient’s quality of life (MLwHF score) was markedly improved after 1 month. In addition, decreased LV end-diastolic and end-systolic volumes, and lower circulating IL-6 levels were observed after 3 months (Schwartz et al. 2008). A phase II study was then conducted in a single arm with open labels with 32 patients using CardioFit. The average intensity was 4.1 ± 1.2 mAmps and patients were observed for 6 months, with a 1-year optional follow-up. Investigators noted that the baseline heart rate decreased significantly over the course of the study suggesting less strain. Additionally, the quality of life (MLwHF score), and the measured 6-min walk test (a test of exercise tolerance in patients with cardiopulmonary diseases), markedly improved at 3 months. They also observed a significant reduction in the LV (left ventricle) end-systolic volume and an increase in the LV ejection fraction, suggesting improved ventricular function. However, this study was small and lacked proper blinded controls (De Ferrari et al. 2017).

The ANTHEM-HF trial was a multi-center open label study to test the safety and efficacy of conducting VNS on either the right or left side along with a continuous cyclic stimulation of 1.5–3.0 mAmps. Left or right side device placement ﻿(Demi- pulse Model 103 pulse generator and PerenniaFLEX Model 304 lead; Cyberonics, Houston, Texas) was randomized among the 60 patients, who still received pharmacological therapy. The researchers noted that there was no difference in safety of the device implantation between the left or right side. There was a significant increase in LV ejection fraction across the whole cohort. Quality of life (MLwHF score) and the 6-min walk score also showed significant improvements after 6 months. It was noted that there was a trend towards right side stimulation being slightly more beneficial than the left, but this was not statistically significant (Premchand et al. 2014).

The INOVATE-HF trial was an 85-center randomized open-label trial with over 700 patients diagnosed with chronic heart failure and an ejection fraction of less than 40%. Patients were randomly assigned to VNS via the same CardioFit implantable stimulator (3.5 to 5.5 mAmps as the target) plus continued medical therapy, or medical therapy alone. This trial concluded that VNS did not increase survival after HF, however it did increase quality of life (KCCQ score) and improved the 6-min walk test with patients being followed up to 4.3 years post VNS implant which is longer than any of the other previous studies (Gold et al. 2016).

The NECTAR-HF trial tested right VNS only with patients randomized to receive either active VNS device or an inactive VNS device. Of the 96 patients, 63 were randomized to the VNS-on and 32 to the VNS-off with the stimulation set at 4 mAmps. This study did not observe any heart rate changes during the first 6 months. Survival at 18 months was not statistically significant between the two groups but left ventricle function improved in the VNS-on group (De Ferrari et al. 2017). Overall, the clinical trials using VNS in heart failure demonstrated some promising results, however the lack of proper controls, as well as the differences in stimulation strengths and durations between the trials, indicates that more studies are warranted to fully analyze the therapeutic potential of VNS. More importantly, the recent studies that demonstrate a vital role for cholinergic signaling in regulating heart regeneration at the molecular level underscores the importance of understanding the mechanisms by which cholinergic signaling mediates heart repair and remodeling following injury (Fig. 1b).

## Conclusions

Nerve-guided tissue regeneration has been demonstrated to be evolutionarily conserved across species, but we are just now starting to understand the role of nerves during mammalian heart regeneration. Our goal in this review is to provide an overview of how cholinergic nerves function during heart regeneration. It remains unclear why the cardiac regenerative window in the mammalian heart is restricted to the early postnatal age. One potential shift during development is the difference between the macrophage compositions of the inflammatory response following injury. We highlight the intersection between cholinergic nerves and the immune response, as inflammation is a critical component of a proper cardiac regenerative response. Dissecting the molecular mechanisms of the cholinergic anti-inflammatory pathway following cardiac injury will help elucidate the cholinergic nerve regulation of the immune response. Cholinergic nerve activation can be achieved with vagus nerve stimulation (VNS), which has already been explored in humans for heart failure treatments. The different results between these studies largely stems from the lack of understanding of the mechanisms by which VNS exerts its function. This underscores the importance of analyzing the molecular effects of VNS on the heart following injury in adult mice, which will guide future clinical investigation and optimization of vagus nerve stimulation. In Fig. 1b, we summarize the potential role of cholinergic nerve stimulation by VNS in guiding mammalian heart regeneration by promoting cardiac repair and regulating the inflammatory response following injury.

Finally, the current rise in aging populations presents a major health care challenge due to the increase in the number of patients with cardiovascular diseases. The emerging role of cholinergic nerve function in regulating heart regeneration provides us with a unique approach to develop novel therapies for heart disease. Unraveling the molecular underpinnings by which cholinergic nerves mediate heart regeneration is an important frontier against the battle to overcome cardiovascular diseases.

## Data Availability

Not Applicable.

## Abbreviations

CM: Cardiomyocyte
ECM: Extracellular Matrix
HF: Heart Failure
KCCQ: Kansas City Cardiomyopathy Questionnaire
LV: Left Ventricle
MHC: Major Histocompatibility Complex
MI: Myocardial Infarction
MLwHF: Minnesota Living with Heart Failure Questionnaire
nAChR: Nicotinic Acetyl Choline Receptor
nAG: Newt Anterior Gradient Protein
NGF: Nerve Growth Factor
NRG1: Neuregulin-1
VNS: Vagus Nerve Stimulation
